# CasanovoGUI: a cross-platform desktop application for deep learning-based *de novo* peptide sequencing with Casanovo

**DOI:** 10.64898/2026.07.11.737889

**Published:** 2026-09-12

**Authors:** Bo Wen, Kai Li, Michael Riffle, Michael J. MacCoss, Wout Bittremieux, William Stafford Noble

## Abstract

*De novo* peptide sequencing detects peptides directly from tandem mass spectra without a protein sequence database, and deep learning has substantially advanced its performance. Casanovo, one such widely used model, is distributed as a Python command-line program. Consequently, installation, GPU and dependency configuration, and manual parameterization can be challenging for many bench scientists and are a recurring source of errors. Interpreting and validating the resulting predictions poses a further challenge. We present CasanovoGUI, an open-source Java-based desktop application that makes all of Casanovo's main analysis functions available through a point-and-click interface on Windows, macOS, and Linux. On first use, CasanovoGUI automatically installs a private Python environment and Casanovo with a GPU-matched build, requiring no prior software setup. The GUI provides access to Casanovo's analysis functions and configuration parameters, streams live progress, and integrates results interpretation: annotated spectra with per-residue confidence scores in the PDV viewer, and mismatch-tolerant mapping of *de novo* peptides back to a reference proteome. CasanovoGUI is available at https://github.com/Noble-Lab/CasanovoGUI.

## Full Text Availability

The license terms selected by the author(s) for this preprint version do not permit archiving in PMC. The full text is available from the preprint server.

